# Addressing key gaps in implementation of mosquito larviciding to accelerate malaria vector control in southern Tanzania: results of a stakeholder engagement process in local district councils

**DOI:** 10.1186/s12936-021-03661-x

**Published:** 2021-03-02

**Authors:** Salum A. Mapua, Marceline F. Finda, Ismail H. Nambunga, Betwel J. Msugupakulya, Kusirye Ukio, Prosper P. Chaki, Frederic Tripet, Ann H. Kelly, Nicola Christofides, Javier Lezaun, Fredros O. Okumu

**Affiliations:** 1 Environmental Health and Ecological Sciences Department, Ifakara Health Institute, P. O. Box 53, Morogoro, Tanzania; 2 School of Public Health, Faculty of Health Sciences, University of the Witwatersrand, Johannesburg, South Africa; 3 School of Life Science and Bioengineering, The Nelson Mandela African Institution of Science and Technology, P. O. Box 447, Arusha, Tanzania; 4 President’s Office-Regional Administration and Local Government, Morogoro Regional Secretariat, P.O. Box 610, Morogoro, Tanzania; 5 Centre for Applied Entomology and Parasitology, School of Life Sciences, Keele University, Huxley Building, Keele, Staffordshire ST5 5BG UK; 6 Department of Global Health and Social Medicine, King’s College London, London, UK; 7 Institute for Science, Innovation and Society, School of Anthropology and Museum Ethnography, University of Oxford, 64 Banbury Road, Oxford, OX2 6PN UK; 8 Institute of Biodiversity, Animal Health and Comparative Medicine, University of Glasgow, Glasgow, G12 8QQ UK

**Keywords:** Malaria control, Malaria elimination, Larviciding, Larval source management, Biolarvicides, Stakeholders, Public perception, Tanzania, Ifakara Health Institute

## Abstract

**Background:**

Larval source management was historically one of the most effective malaria control methods but is now widely deprioritized in Africa, where insecticide-treated nets (ITNs) and indoor residual spraying (IRS) are preferred. However, in Tanzania, following initial successes in urban Dar-es-Salaam starting early-2000s, the government now encourages larviciding in both rural and urban councils nationwide to complement other efforts; and a biolarvicide production-plant has been established outside the commercial capital. This study investigated key obstacles and opportunities relevant to effective rollout of larviciding for malaria control, with a focus on the meso-endemic region of Morogoro, southern Tanzania.

**Methods:**

Key-informants were interviewed to assess awareness and perceptions regarding larviciding among designated health officials (malaria focal persons, vector surveillance officers and ward health officers) in nine administrative councils (n = 27). Interviewer-administered questionnaires were used to assess awareness and perceptions of community members in selected areas regarding larviciding (n = 490). Thematic content analysis was done and descriptive statistics used to summarize the findings.

**Results:**

A majority of malaria control officials had participated in larviciding at least once over the previous three years. A majority of community members had neutral perceptions towards positive aspects of larviciding, but overall support for larviciding was high, although several challenges were expressed, notably: (i) insufficient knowledge for identifying relevant aquatic habitats of malaria vectors and applying larvicides, (ii) inadequate monitoring of programme effectiveness, (iii) limited financing, and (iv) lack of personal protective equipment. Although the key-informants reported sensitizing local communities, most community members were still unaware of larviciding and its potential.

**Conclusions:**

The larviciding programme was widely supported by both communities and malaria control officials, but there were gaps in technical knowledge, implementation and public engagement. To improve overall impact, it is important to: (i) intensify training efforts, particularly for identifying habitats of important vectors, (ii) adopt standard technical principles for applying larvicides or larval source management, (iii) improve financing for local implementation and (iv) improve public engagement to boost community awareness and participation. These lessons could also be valuable for other malaria endemic areas wishing to deploy larviciding for malaria control or elimination.

## Background

The world has witnessed a significant reduction in malaria burden since 2000 [1], most of these gains being attributed to insecticide-treated bed nets (ITNs), indoor residual spraying (IRS) and effective case management [2, 3]. Yet, there were still more than 200 million cases, and 405,000 deaths globally in 2018, 90% in sub-Saharan Africa [1]. Ongoing malaria control efforts are increasingly compromised by several factors, chief among them, parasite resistance to anti-malarial drugs [4, 5], behavioural adaptation of mosquitoes to ITNs and IRS [6, 7] and growing insecticide resistance in malaria vectors [8, 9]. Anthropological factors also play a crucial role in mediating transmission, as human behaviours, economic practices and perceptions of risk can increase dangers of infectious malaria vectors [10–13]. Malaria vector control in Tanzania has also focused mainly on provision and use of ITNs and IRS [14–18]. This is complemented with other efforts such as increased access to reliable and affordable diagnostics and treatment [19], and universal distribution of prophylaxis for pregnant women [20]. These efforts, combined with a general improvement in economic opportunity, have led to a tremendous decline in malaria burden throughout the country [20, 21].

Environmental management to eliminate mosquito breeding habitats was among the first malaria control strategies attempted in Tanzania. Efforts included improving drainage systems and the elimination of the permanent bodies of stagnant water near large human settlements [22, 23]. In recent times, the first major use of larviciding in Tanzania was in Dar-es-Salaam in early 2000s [24, 25], when regular application of biolarvicides by community-owned resources persons (CORPs) achieved as much benefit as ITNs [25].

The Tanzania National Malaria Strategic Plan, 2014–2020 recommended implementation of larviciding in selected urban settings [26], in line with guidance from the World Health Organization to consider only settings where aquatic habitats of malaria vectors are few, fixed and findable [27]. This policy initially focused on just urban populations, but in recent years the government has encouraged extension of larviciding to include rural settings [28].

The nationwide expansion of larviciding follows the creation in 2014 of Tanzania Biotech Products Limited (TBPL), which is responsible for production and distribution of biolarvicides [29]. Since 2017, TBPL has been manufacturing two types of biolarvicides, *Bacillus thuringiensis* var. *israelensis* (*Bti*) and *Bacillus sphaericus* (*Bs*) [29]. These products are procured by the district councils across the country, and distributed to all administrative wards. Councils often reserve budgets to compensate community-health workers (CHWs) and volunteers involved in community initiatives such as larviciding [30].

The recent developments by Tanzania to expand larviciding are excellent examples of the much-needed ownership for sustainable vector control, especially given the use of the domestic resources. If sustained, it could yield significant gains over current accruals from the core interventions, and in the process generate important lessons for other countries. Unfortunately, given its extensive scale and novelty as well as the inclusion of predominantly rural councils, there are still multiple challenges that must be addressed to achieve maximum impact. For example, the major malaria vectors in the country use a wide variety of aquatic habitats, which still need to be sufficiently characterized [31]. Moreover, larviciding is also labour-intensive and requires active community involvement.

This study, therefore, aimed to identify and characterize important gaps as well as opportunities for improving the implementation of larviciding in Tanzania. The study examined perceptions and experiences of key actors of larviciding in different district and municipal councils. The study focused on the mostly meso-endemic region of Morogoro, southeastern Tanzania.

## Methods

### Study area

**Fig. 1:**
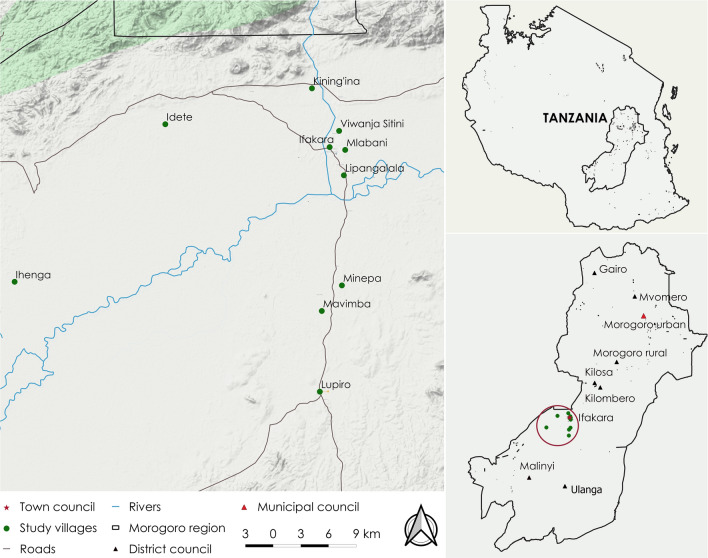
Map of Morogoro Region, Tanzania, showing the districts, wards and villages where the study was conducted. Map prepared by Najat Kahamba

The study was conducted in nine administrative councils in the Morogoro region in southern Tanzania between October 2019 and March 2020 (Fig. 1). The area has a total population estimated at 2.2 million people [32], and is currently classified as meso-endemic, with malaria prevalence estimated at ~ 10% according to the most recent estimates [33]. The councils were: Gairo, Mvomero, Kilombero, Ulanga, Kilosa, Morogoro and Malinyi district council, Morogoro municipal council and Ifakara town council (Fig. 1). The community members surveyed were from Ulanga and Kilombero districts only.

### Selection of stakeholders

Stakeholders selected for this study included district health officials and community members. The health officials included district malaria focal persons (MFPs), vector surveillance officers (VSOs) and ward health officers.

Malaria focal persons were either medical doctors or environmental health specialists in charge of all malaria related-matters at the district level. In this study, all the MFPs had been at their current position for at least two years. They are responsible for all aspects of malaria control, including monitoring trends of malaria cases, deaths and control. Vector surveillance officers on the other hand were environmental health specialists with a diploma in environmental health science and a special training in disease-vector control. The VSOs are responsible for organizing, supervising and executing disease-vector control programmes at the district level. Lastly, the ward health officers were also environmental health specialists and were responsible for all health-related issues at the ward level. They had a diploma or certificate training in environmental health science, and their responsibilities included planning, supervising, monitoring and evaluating overall health services at the ward level. Each district has one MFP, one VSO and multiple ward health officers, but in some cases one ward health officer could serve multiple wards within the district.

Malaria focal persons and VSOs were recruited from all districts as well as the municipal and town councils within Morogoro region. However, the ward health officers were recruited from a randomly selected ward in each district, municipal or town council. Each of seven districts had between 8 and 38 wards.

For the community survey, households were randomly selected from ten randomly selected wards in Ulanga and Kilombero districts in the region (Fig. 1), and the survey was administered to the household heads.

### Study design and procedures

A concurrent triangulation mixed method study design was used [34], incorporating key informant interviews (KII) and survey questionnaires. Key informant interviews were done with MFPs, VSOs and ward health officers to obtain information on the degree of awareness as well as experiences and perceptions of these officers regarding larviciding. These interviews were conducted by the authors, SAM, MFF and IHN, between February and March of 2020 at the respective council offices. The interviews were audio-recorded following consent of the participants. The audio recordings were supplemented by hand-written notes. Each interview lasted between 15 and 60 min and were done in Swahili language.

The questionnaire surveys were conducted with community members from Ulanga and Kilombero district. These were done in Swahili language, and used to gather data on awareness and perceptions of larviciding as a malaria control intervention. Kobotoolbox™ software [35] was used to administer the surveys via electronic tablets, between November and December 2019. The individual-level perception of community members towards larviciding was assessed by measuring the level of agreement towards positive statements on larviciding using a 5-point Likert-scale, ranging from strongly agree (1) to strongly disagree (5). The main statements were as follows: (i) larviciding will be effective for malaria control, (ii) larviciding will fill gaps left by other interventions, (iii) larviciding is safe for humans, animals and the environment, (iv) larviciding will be easy to perform, (v) larviciding supplies and equipment will be easily accessible, (vi) larviciding will be affordable to community members and (vii) larviciding will be acceptable in the community. The final perception level was determined by comparing individual perception scores against the median score (see “Data processing and analysis” section).

In addition, one joint stakeholder engagement meeting was conducted at the regional office, where all the MFPs and VSOs from the nine administrative councils participated, together with Ifakara Health Institute researchers. Discussions at this meeting involved options for improving larviciding operations in the respective councils, and what roles different stakeholders could play.

### Data processing and analysis

Audio recordings of the key informant interviews were transcribed immediately following the discussions and translated from Swahili to English language. Field notes were added in the written transcripts. The written transcripts were analysed using NVIVO 12 Plus software [36]. Deductive and inductive coding were used to categorize the codes items. A KII guide was used to develop the deductive codes while the inductive codes were generated based on thorough reviews of the transcripts. Similar codes were grouped and emergent patterns used to identify themes. The extracted themes included: (i) knowledge about larval habitats of malaria vectors, (ii) awareness of larviciding as a malaria control intervention and (iii) challenges facing the implementation of larviciding. Direct quotation from participants were used to support the themes. Information from the key informant interviews and survey were triangulated during the discussion of the findings [37].

The quantitative data on the other hand was analysed using R statistical software version 4.0.0 [38]. The sum of the scores of the seven statements was calculated for each survey respondent, after which a median of these scores calculated. Perception level was determined by comparing individual perception scores against the median perception score; scores above the median were considered negative while those at or below the median were considered positive. Internal validity of the scale was measured by calculating Cronbach’s alpha [39]. Univariate analysis was used to determine influence of the respondent sex, age group, education level and degree of previous awareness of larviciding on the main outcome variable, i.e. their perceptions of larviciding. Binary logistic regression was used to determine the association between the independent variables and the outcome variable; odds ratio was calculated at 95% confidence intervals (CIs).

## Results

### Characteristics of study respondents

A total of 517 people (43% men and 57% women) participated in this study. These included the 27 key informants who participated in the in-depth interviews, and 490 community members responding to the administered questionnaires. Nineteen of the 27 KII participants were men, and all participants had a college or university degrees. The average age of participants in KII was 45 years, ranging from 33 to 60 years. Average duration of employment in their current position and at their current location was 7 years, ranging from 6 months to 35 years (Table 1).

**Table 1:** Characteristics of Key Informant Interviewees

Key informants	Mean age (years)	Average no. years in service	Males	Females	Total
Malaria Focal Persons	40.1	4.5	6	3	9
Vector Surveillance Officers	47.9	7.4	6	3	9
Ward Health Officers	47.2	9.2	7	2	9
All Participants	45.1	7.0	19	8	27

Average age of the community members who participated in the survey was 42 years (range: 18–88 years) and two thirds (66%, n = 321) were married. About three quarters (73.1%, n = 358) had primary school education, 8.8% (n = 43) had no formal education, 13.9% (n = 68) had secondary education and 4.3% (n = 21) had college-level education. A majority (84.3%, n = 413) of the respondents reported small-scale farming as their main income-generating activity, but people also practiced small retail businesses, fishing, animal husbandry or had formal employment.

### Perception regarding malaria burden

Table 2 summarizes the respondent perceptions regarding malaria burden in Tanzania. Nearly a half of the survey respondents reported not knowing the current malaria prevalence range in Tanzania. Only 15.3% identified correct range of nation-wide prevalence (6–10% based on 2018 Malaria Indicator Survey [33]). Two thirds believed that rural communities or poor households suffer the heaviest burden. More than a half of respondents believed the country was progressing well towards elimination, and that it could achieve elimination with current interventions. However, a majority of the survey respondents noted that alternative interventions would be necessary to speed up these efforts (Table 2).

**Table 2:** Community perceptions regarding malaria risk and burden (N = 490)

Questions asked	Variables	Percentage (n)
Which settings are at highest risk of malaria?	Rural settings	65.1 (319)
	Urban settings	7.6 (37)
	Equal in rural and urban settings	23.7 (116)
	Do not know	3.7 (18)
Which communities are most affected by malaria?	Low-income communities	63.9 (313)
	All communities are equally affected	33.7 (165)
	Do not know	2.5 (12)
Where does most malaria transmission occur?	Outdoors	61.3 (300)
	Indoors	36.7 (180)
	Do not know	2.0 (10)
What is your opinion regarding country’s progress towards malaria elimination	Very good	51.6 (253)
	Good but slow	43.9 (215)
	Very slow	4.5 (22)
Can malaria be eliminated	Possible	59.6 (292)
	Not possible	40.4 (198)
Do we need alternative interventions?	There is a need	86.1 (422)
	No need	13.9 (68)

### Awareness of community members regarding larviciding as a malaria intervention

Only a quarter of survey respondents were aware of the government policy to include larviciding as a malaria intervention (Table 3), and more than half did not know whether the intervention was ongoing in their districts. Three quarters also did not know the mode of action of larvicides despite knowing what the intervention itself is. Older respondents (46–55 years) were more aware of larviciding than those 25 years or younger.

**Table 3:** Knowledge and awareness of larviciding in the communities (N = 490)

Variable assessed	Response	Percentage (n)
Awareness of larviciding (n = 490)	Yes	26.1 (128)
	No	73.9 (362)
Sources of information (n = 128)	Friends/family	48.1 (76)
	Radio/TV	21.5 (34)
	IHI scientists	10.8 (17)
	Community meetings	7.6 (12)
	Saw on a visit in Dar es Salaam	7.6 (12)
	Community health workers	4.4 (7)
Has larviciding been implemented in the community (n = 490)	Yes	4.5 (22)
	No	43.5 (213)
	Do not know	52.2 (255)
Larviciding works by killing mosquitoes in their juvenile stage (n = 490)	Agree	23.9 (117)
	Do not agree	2.0 (10)
	Do not know	74.1 (363)

### General perception of larviciding and its potential as a malaria intervention

Perception of community members towards larviciding was assessed based on levels of agreement towards positive statements on a 5-point Likert-scale, ranging from strongly agree to strongly disagree. The median score of the seven statements was 21. Reliability assessment of the perception scale yielded a Cronbach alpha score of 0.77, indicating acceptable reliability of the scale and minimum redundancy.

Of all survey participants, 40.4% agreed that larviciding would be acceptable in their community as new intervention. The rest of the community members had neutral perceptions on effectiveness, safety, feasibility, accessibility, affordability or acceptability of larviciding (Table 4). Community members who were already aware of larviciding were more likely to welcome larviciding compared to respondents without previous knowledge prior to the survey (p = 0.029), Table 5). However, three quarters (74.2%, n = 364) of respondents said they would support larviciding if introduced to their communities.

**Table 4:** Perception of community members regarding effectiveness, feasibility, affordability and acceptability of larviciding for malaria prevention (N = 490)

Statement	Strongly agree (1) (%)	Agree (2) (%)	Neutral (3) (%)	Disagree (4) (%)	Strongly disagree (5) (%)
Will be effective	29.8	14.7	54.5	0.4	0.2
Will fill gaps left by ITNs	28.4	13.1	56.1	1.2	1.2
Will be safe for humans, animals and environment	7.1	8.4	76.9	3.9	3.7
Will be easy to use	19.6	4.7	72.5	2.0	1.2
Will be easily accessible	2.6	2.2	84.1	4.1	6.9
Will be affordable to residents	2.9	1.4	86.7	1.6	7.4
Will be acceptable in community	34.3	6.1	56.7	2.2	0.6

**Table 5:** Association between the community perception towards larviciding and their socio-demographic characteristics

Category	Variable	Odds ratio (95% CI)	p-value
Sex	Male	1.00	–
	Female	0.74 (0.32, 1.70)	0.470
Age category (in years)	18–25	1.00	–
	26–35	0.53 (0.14, 2.58)	0.382
	36–45	0.56 (1.34, 2.76)	0.428
	46–50	0.42 (0.07, 2.36)	0.300
	Above 50	0.60 (0.14, 3.04)	0.497
Education level	No formal education	1.00	–
	Primary (7 years)	2.09 (0.41, 38.20)	0.478
	Secondary (12 years)	1.94 (0.24, 39.90)	0.752
	Tertiary (> 12 years)	7.00 (0.83, 146.87)	0.102
Awareness of larviciding	Aware	1.00	–
	Not aware	0.40 (0.17, 0.93)	0.029*

The odds and p values represent likelihood of certain groups having a favourable opinion of larviciding as a malaria intervention

*Statistically significant difference

### Awareness, perceptions and experiences of district and ward-level health officials regarding larviciding for malaria control

#### Important aquatic habitats of malaria vectors

In the initial analysis, most KII participants reported that they knew the general characteristics of mosquito aquatic habitats, but not all were able to distinguish between habitats of key malaria vectors and habitats of other mosquitoes. When asked to describe the aquatic habitats of important malaria vectors, respondents used terminologies such as fresh waters, standing waters, pit latrines, trash pits, septic pits, used tires, long grass and bushes.

When considered separately, most malaria focal persons and vector surveillance officers were able to distinguish between aquatic habitats of malaria vectors. They pointed out that *Anopheles* mosquitoes prefer fresh water. A small number of MFPs however were unable to make this distinction, despite knowing that some mosquitoes preferred fresh water. They were unable to specify key characteristics of the actual malaria vectors as distinguishable from the habitats of non-vectors. On the other hand, a majority of the ward health officers were not aware of the differences in breeding habitats between malaria and non-malaria vectors. This group only knew that mosquitoes breed in water. They identified ponds, streams and river banks, septic tanks and pit latrines as possible breeding habitats for all mosquitoes. They conceded that differentiating larval habitats was too technical a task for their capacities; their focus was on identifying places with standing water and treating them with larvicides.

*“It is not too easy to differentiate between the larval habitats, except if you see a place with a lot of water, then you just know that there will be mosquito larvae there, because we know mosquitoes like to lay their eggs in water. In my ward, for example, we have water ponds that last a whole year, so I know mosquitoes breed there. There are also communities where people still use pit latrines, but the holes are not covered and the toilets do not have doors or roofs. So I also know that mosquitoes can breed in those.”* (Ward Health Officer, Male).

The term ‘fresh water’ generated great discussion among the key informants. Those who reported that malaria vectors preferred clean and fresh water also listed water storage buckets or pots and morning dew as potential habitats for malaria vectors.

*“What I know is that there are different types of mosquitoes; I know there are Anopheles, Culex and Aedes mosquitoes. I know that Anopheles prefers to breed in clean and fresh water, so they can be found in buckets of clean water, in the clean morning dew. Culex on the other hand likes dirty water; they like to lay their eggs in septic pits and in other dirty places*.” (Vector Surveillance Officer, Male).

#### Knowledge of larviciding

 All MFPs, VSOs and ward health officers knew that larviciding involved killing mosquitoes with chemicals during their larval stages. They also knew of two types of biolarvicides (i.e. *Bti* and *Bs*) available for large-scale implementation in Tanzania, one used to treat fresh and clean water, and the other one used to treat dirty water. Many could however not name the biolarvicides, nor specify which types were applicable for malaria-vector control.

*“Larviciding it is the killing of the second stage of mosquito’s life cycle using chemicals called larvicides. In Tanzania we have biological larvicides, so they are called biolarvicides. I understand that these biolarvicides are some kind of bacteria; when they are put in water that contains mosquito larvae, the larvae feed on the bacteria, which kills them.”* (Malaria Focal Person, Male).

#### Supply and distribution of larvicides

 MFPs reported having received two types of biolarvicides (totaling 720 l per council) from the government to distribute to the wards within their districts through ward health officers. The first supply was delivered in 2018, and another supply delivered in 2019. It was noted that the distribution of the biolarvicides had been prioritized on wards with the highest reported malaria cases compared to others.

#### Implementation of larviciding

 To support larviciding, the ward health officers recruited and trained community health workers (CHW), local residents who had previously participated in a community health training course. Where no CHWs were available, the ward health officers recruited volunteers, who were typically young male residents. The CHWs or volunteers were responsible for actual application of larvicides, with supervision from the ward health officers. The ward health officers would accompany the implementers to identify water bodies within their wards and during the first application. Unfortunately, a majority of the ward health officers had received no specific training on how to implement the larviciding. Moreover, in some districts one ward health officer was responsible for overseeing larviciding in up to four wards, thus they were unable to effectively supervise the CHWs.

*“I supervised this work throughout. I recruited community health workers from different communities in my ward and gave them larvicides. This way I made sure that every community in my ward had larvicides.”* (Ward Health Officer, Male).

*“We were told to involve the community when we received the larvicides, so we spoke with village and community leaders, and with their help we found young men in the communities to help with this work. We then instructed the young men on how to apply the larvicides.”* (Ward Health Officer, Male).

#### Training on application of bio‐larvicides

 Malaria focal persons reported that they had participated in at least one seminar on how to apply the larvicides, in 2018 and or 2019. Some of the MFPs were not holding their current positions in 2018 and had therefore only received one training session. The training, provided jointly by the Muheza College of Health and Allied Sciences [40] at Muheza district and Kibaha Biotech Products Limited (TBPL) [29], was described as largely theoretical, providing information on the two types of biolarvicides and where to use them. There had been no practical training on identification of aquatic habitats, application of larvicides or monitoring of programme effectiveness. Fortunately, all MFPs had been given written guidelines for biolarvicides application.

*“I participated in this year’s [2019] seminar. We were given a formula on how to calculate the amount of larvicides per liter, and they promised to share with us the template with the specific formula for the amount of diluted larvicides to apply in a breeding habitat. It was a PowerPoint presentation; it was all theoretical.”* (Malaria Focal Person, Male).

Unlike the MFPs, the VSOs and ward health officers reported not to have participated in the training programmes, but had instead received information on dilution and application methods from the MFPs. Ward health officers then passed on the information to the CHWs and the community volunteers who were responsible for the hands-on implementation of the larviciding.

*“I called the volunteers to my office and explained how to dilute the larvicides and how to apply them to the breeding habitats. I did the training in my office. Then I provided them with the larvicides as well as masks to protect themselves.”* (Ward Health Officer, Female).

#### Monitoring efficacy of the larvicides

 There was no formal mechanism of monitoring effectiveness of the larviciding. Some ward health officers stated that they kept track of the number of malaria cases at the health centers, and assumed that reduced cases meant that the larviciding was working. Other ward health officers reported that they asked community members if they had experienced a reduction in mosquito annoyance. Others relied on their own experience living in the communities to detect a reduction in mosquito abundance. All respondents reported that they believed that larvicides were effective based on these factors.

#### Challenges during implementation of larviciding

 Key challenges that district and ward health control officers faced during implementation of larviciding are summarized on Table 6 below. The challenges listed included insufficient technical knowledge on identifying habitats of malaria vectors and application of the larvicides, insufficient knowledge on safety of the larvicides, inadequate funding, inadequate supply of larvicides, some resistance from community members, late-involvement of VSOs and ward health officers and inadequate collaboration from non-governmental organizations in the districts or wards.

**Table 6:** Key challenges facing larviciding programs in Morogoro region, southern Tanzania

	Challenges	Description	Examples of respondent quotes
1	Insufficient technical knowledge on habitat identification and larviciding	Malaria Focal Persons, District Surveillance Officers and Ward Health Officers reported that they did not have adequate technical knowledge for assessing whether specific water bodies were likely to contain mosquito larvae, and whether those larvae were likely to belong to *Anopheles* species or other mosquitoes. As a result, ward health officers reported that they often treated all the water bodies they could find in their wards The MFPs also reported that they did not have accurate information on the proper amount of larvicides to apply in specific water bodies. Instead, they often just guessed the amount, based on their perceived volumes of the habitats There was also no uniformity on methods of monitoring efficacy of the larvicides. Some reported that they used number of malaria cases at the health centers as an indicator of efficacy and some used community testimonials on reduced mosquito nuisance bites	*“it is not easy to differentiate mosquito breeding sites, however, there are areas that you can recognize as breeding sites upon seeing. For example, we have areas with ponds that last the whole year and a great example is an area close to the secondary school where brick laying created ponds which obvious attract mosquitoes as a breeding site.”* (Ward Health Officer, Male) *“Like I said, we lack knowledge on this aspect. We do not even know how much larvicides to spray in a water pond for example. Even if you ask the VSO he will tell you the same. So then we do a lot of guess work, but we do not know for sure if we are putting too much or too little.”* (Malaria Focal Person, Female) *“We do monitoring by asking community members, they are the ones who report sleeping comfortably.”* (Ward Health Officer, Female) *“We look at the statistics, as to whether number of malaria patients increasing or decreasing.”* (Ward Health Officer, Female)
2	Lack of knowledge regarding safety of the larvicides	There were also inconsistencies in knowledge about risks posed by the larvicides. MFPs and VSOs claimed that the larvicides did not pose any harm to people or their livestock, but were not sure whether the larvicides could cause harm to other aquatic organisms. In contrast, most ward health officers believed the larvicides could harm people or animals, since they smelled like poison and turned the color of the water	*“I know that it is safe on humans, but I really do not know if they pose any harm on other insects in the water, on animals or on vegetation around the water. I only know that it does not have any harm on humans.”* (Malaria Focal Person, Male) *“It has to have harm, I can just tell from the smell that comes when you apply it, the water also turns milky, so it just looks poisonous. So I advise people to not use the water immediately after the application, but if they wait after a while the smell disappears and the color goes back to normal.”* (Ward Health Officer, Female)
3	Inadequate funding	All participants reported that lack of sufficient funding was a significant obstacle for successful implementation of larviciding. Funding was needed to provide compensations and wages to the CHWs or the volunteers, procure personal protective gear and application equipment and for transportation In some cases the participants reported limiting larviciding activities due to limited financial support	*“When you ask people in the community to help with this exercise, they expect to get a wage. But when we were implementing this there wasn’t any money set aside for paying the volunteers or the CHWs. Sometimes I had to give them my own money, because I saw how hard they were working.”* (Ward Health Officer, Male) *“In my district we had to stop before finishing because we just did not have any money to implement this project. We had the larvicides only, but nothing else. We requested money for protective gear, transportation, or for paying people that were doing the application but we did not receive it, so after some time we just had to stop.”* (Vector Surveillance Officer, Female) *“For an example, my district has 31 wards, and it is not like the breeding habitats are at the headquarters of the wards. You have to go deep into the villages. It is hard to walk with a can containing 20-liters of larvicide. There is only one car at the district, and even that is currently not functioning.”* (Vector Surveillance Officer, Male)
4	Inadequate supply of larvicides:	Some of the ward health officers reported that the larvicides they received were not enough to treat all mosquito breeding habitats in their area of jurisdiction. In particular, communities living in swampy areas, needed a lot more supplies than they received	*“I will tell you that the larvicides were not enough. In all the breeding habitats that I had surveyed, we could not cover all of them before running out of the larvicides. We needed more, but there was none.”* (Ward Health Officer, Female) *“In 2018, I have received two cans of twenty liters which cannot be enough for my ward. In another round, I had received two cans of twenty liters per village which was not enough either, so we decided to prioritize the most significant settings.”* (Ward Health Officer, Female)
5	Some resistance from members of the community	Key informants reported initially facing resistance from some community members who feared that the larvicides would be poisonous to chicken, livestock or fish. This was mostly due to the smell of the larvicides, and by the fact that the water turned milky immediately after application. This initial resistance was however reported to ease once the health officials spent time explaining the benefits and safety of the larvicides. Community sensitization was primarily done by ward health officers with assistance from CHWs	*“The uptake was not very good in the beginning as people were not educated on what larvicides are, how they work or their safety. So they were always reluctant to let people spray near their homes.”* (Vector Surveillance Officer, Female) *“Once people were sensitized, the uptake improved. People would even follow us and ask when we would be spraying again, or point me to breeding habitats that I had missed.”*(Ward Health Officer, Male)
6	Inadequate involvement of VSOs and Ward health officers in early stages	VSOs and ward health officers reported to not being involved in the initial planning of the larviciding programme at the district level, but rather receiving implementation plan from malaria focal person. This overshadows their significant inputs as they have spent more time in the settings on average compared to malaria focal persons	*“I was not involved in the planning and these larvicides are new which requires training but we have only been given pamphlets. Only if we can be involved from the early stages, I think it will improve the practice.”* (Vector Surveillance Officer, Female)
7	Insufficient collaboration with non-governmental organizations	Key informants reported inadequate involvement of the non-governmental organizations (NGOs) in the implementation of the larviciding programme. This has been attributed to larviciding not being priority among these NGOs	*“Providing awareness to the community, maybe we could try but even Boresha Afya indicated disease prevention is not in their priorities but rather case management. SolidarMed priorities are in behavioural change, so we have no stakeholders in disease prevention.”* (Malaria Focal Person, Male)

The table provides a brief description of each identified challenge, as well as examples of direct statements from the study respondents

## Discussion

Larviciding is considered as complementary option to be used alongside current major malaria control approaches, notably ITNs, IRS and case management [41]. To accelerate malaria elimination efforts, the Tanzanian government has invested significantly in larviciding, including the establishment of a national production capacity and adoption of larviciding in both rural and urban settings [26]. This study investigated some of the practical obstacles that limit the effective roll-out of this strategy across the country, with a particular focus on the perceptions and experiences of key stakeholders of malaria control in southern Tanzania.

The key-informant interviews revealed significant knowledge inadequacies among MFPs, VSOs and ward health officers towards implementation of the larviciding. For instance, all participants knew that mosquitoes have an aquatic habitat stage; but a majority could not easily differentiate the aquatic habitats typical of malaria vector species. Moreover, these health officials reported that malaria vectors do prefer “fresher” water compared to other mosquitoes, but what majority meant by fresh water was any water that looked clean such as water in clay pots or buckets. Ward health officers, who are closely anchored in the community and provide guidance to the community health workers and volunteers during the larviciding, could not differentiate between malaria and non-malaria vectors’ aquatic habitats and reported to use different methods to apply and monitor effectiveness of the larvicides. This lack of adequate knowledge and uniformity might be attributable to the lack of training on how, where and when to apply the larvicides as accorded by WHO guidelines [41]. Some of these malaria control officials particularly MFPs and VSOs reported to have attended at least one theoretical training on larviciding. However, this training proved to be insufficient as acquiring necessary expertise would require practical, “on the job” training rather than a presentation of theoretical principles [42]. No formal training to the actual implementers (i.e. ward health officers, CHWs and volunteers) was reported, this could undermine the overall impact of the programme.

Insufficient funding to assist with implementation of larviciding was one of the practical obstacles reported by the MFPs, VSOs and ward health officers. Funding was needed to offer incentives, cover transportation and larvicides costs, and provide personal protective gears to the CHWs and volunteers who did the actual job of applying the larvicides. A successful large-scale larviciding trials conducted in Dar-es-Salaam [25, 43] in early 2000s had demonstrated the cost-effectiveness of the approach [44]. However, larviciding is deemed operationally and financially infeasible in the rural settings [41]. Fortunately, a recent study by Nambunga et al. in rural Tanzania highlighted the possibility of minimizing the unnecessary costs, if larviciding could be species-specific [31].

In Kilombero valley, *Anopheles funestus* accounts for over 80% of the ongoing malaria transmission [8], its aquatic habitats have found to be few and highly distinctive [31]. Thus, effective targeting of *An. funestus* aquatic habitats alone could potentially reduce malaria transmission by 80% in Kilombero valley. In this valley, *An. funestus* aquatic habitats adhere to WHO criteria (i.e. few, fixed and findable) for larviciding implementation [41]. The application of larvicides for malaria control in Morogoro region is often directed towards all stagnant water bodies, thus undermining the intended amount of larvicides. Understanding the ecology of major malaria vectors in each district within Morogoro region could cut the unnecessary costs and provide effective larviciding approach. However, studies shows that control of Culicine mosquitoes that are responsible for enormous biting nuisance could maximize community acceptance and support towards malaria control programme [45, 46].

This present study also revealed the need to strengthen engagement of key stakeholders including the community. Despite efforts by district-level malaria control officials to inform and sensitize the residents, a majority of the community members surveyed were not aware of larviciding, did not know its function within malaria control efforts, and were not aware whether or not it had been implemented in their settings. This finding was in agreement with a previous study by Mboera et al.. in Mvomero district within Morogoro region, where only 17% of the survey respondents were aware of larviciding as a malaria control intervention [47]. Both findings indicate inadequate community engagement methods during the implementation stage. However, community members in both studies showed willingness to support the implementation of larviciding in their communities. In the present study, age, gender and educational level of the survey respondents did not seem to influence their level of awareness and perception towards larviciding, but the contrary was observed in other studies [48, 49]. The majority of the districts in Morogoro region have at least one local radio station, which may be relied upon to further strengthen the community engagement.

Insufficient support from local stakeholders within Morogoro region might have been among the obstacles towards effective implementation of larviciding. Engagement of other stakeholders particularly non-government organizations (NGOs) have shown to yield fruitful impact in the malaria control. For instance, collaboration between Urban Malaria Control Programme (UMCP) and Ifakara Health institute (IHI) in Dar-es-Salaam during early 2000s towards malaria control through larval source management led to a significant impact [25]. Thus, effective engagement of these NGOs such as IHI will somewhat ensure smooth implementation of larviciding through resources provision and/or capacity building.

The present study also revealed insufficient “early-on” involvement of VSOs and ward health officers during the budgeting and implementation planning. MFPs attend all council’s meeting that involve malaria control initiatives through district technical committee [50], and often instruct the VSOs and ward health officers on the way forward. This could lower the sense of ownership towards the larviciding programme. Adequate involvement of VSOs and ward health officers could strengthen the implementation of the programme, apart from VSOs holding a special training on disease-vectors control but also majority have spent significant number of years in the localities.

The study results should be interpreted in the light of several limitations. A response bias may have resulted partially inaccurate responses on the survey. Social desirability bias may have resulted in respondents saying ‘I don’t know’ to most of the statements that assessed their perceptions of larviciding as a majority had early-on indicated that they were not aware of this intervention. Demand characteristics may have also resulted from both the key informants who may have reported insufficient knowledge or lack of resources hoping that these would be provided to them. In addition, the present study did not include district medical officers (DMO) who also plays a crucial role in planning, coordinating and implementing the delivery of health services at the district level [30].

## Conclusions

Both communities and district-level malaria control officials widely supported the larviciding programme, however, there were gaps in technical knowledge, implementation and stakeholder engagement. To maximize the overall impact of the programme, training efforts should be intensified, particularly for identification of aquatic habitats for important vectors and formal training should be given to the actual implementers (i.e. CHWs and volunteers) not just MFPs, VSOs and ward health officers. Standard technical principles for application of larvicides should strictly be adopted and improvement on financing at a district-level implementation. Furthermore, engagement of community members and other stakeholders such as NGOs should be improved to maximize awareness, participation and sustainability of the programme. These lessons learnt from Morogoro region shed the light for other malaria endemic areas on the possibility of deploying larviciding for malaria control or elimination.

## Data Availability

The data will be made available by the corresponding author upon reasonable request.

## Abbreviations

KI: Key informant
KII: Key Informant Interview
MFP: Malaria focal person
VSO: Vector surveillance officer
NMSP: National Malaria Strategic Plan
ITN: Insecticide-Treated bed Net
IRS: Indoor Residual Spraying
*Bti*: *Bacillus thuringiensis *var.* israelensis*
*Bs*: *Bacillus sphaericus*
IHI: Ifakara Health Institute
